# Profilaxis urbana frente al brote de cólera en Arica, Chile, 1886-1888

**DOI:** 10.1590/S0104-59702024000100065

**Published:** 2024-12-16

**Authors:** Pablo Chávez Zúñiga

**Affiliations:** i Investigador, Centro de Estudios Históricos/Universidad Bernardo O’Higgins. Santiago – Chile pablo.chavez.zuniga@gmail.com

**Keywords:** Epidemia cólera, Higiene urbana, Arica, Chile, Siglo XIX, Epidemics cholera, Urban hygiene, Arica, Chile, Nineteenth century

## Abstract

El objetivo de esta investigación es analizar el brote de cólera que se produjo en la ciudad de Arica, situada en la frontera norte de Chile-Perú, a finales de la década de 1880. El estudio se enfoca en el impacto que generan las epidemias en la sociedad, los diagnósticos médicos y la rápida propagación del contagio. Se aborda la creación de una institucionalidad local para enfrentar la emergencia sanitaria y las políticas para evitar la llegada de la afección al puerto. Basándonos en la literatura especializada, la prensa ariqueña y la documentación del Archivo Histórico Vicente Dagnino, concluimos que el brote de cólera activó una serie de mecanismos político-sanitarios que transformaron las prácticas de higiene pública y privada.

## Abstract (en)

The objective of this research is to analyze the outbreak of cholera that occurred in the city of Arica, located on the northern border of Chile-Peru, at the end of the 1880s. The study focuses on the impact generated by the epidemics in society, medical diagnoses and the rapid spread of contagion. The creation of an institutional framework to face the health emergency and the policies that were implemented to prevent the arrival of the condition to the port are addressed. Based on specialized literature, the Arican press and the documentation of the Vicente Dagnino Historical Archive, we concluded that the cholera outbreak activated a series of political-health mechanisms that generated transformations in public and private hygiene practices.

Hemos visto como el cólera morbus, el viajero del Ganges trasmontando las sierras, cruzando desiertos y los mares por vía de comunicación facilísimas sigue infatigable a la especie humana sembrando por todas partes el terror y haciendo su lecho endémico principalmente en aquellas poblaciones desprovistas de higiene.

Este mundo microscópico de seres vivos que avanza rápido en persecución del hombre para destruir su organismo hallaría entre nosotros, duro es decirlo, una morada imperecedera dadas las condiciones anti-higiénicas de nuestra población si con el estudio del clima y modo de ser especial de los pobladores de la localidad no tomamos pronto las medidas preventivas más indispensables (El Porvenir, 1886-1887, [4 ene. 1887]).

Con estas palabras el Comité de Higiene y Salubridad local presentó un estudio al Comité de Higiene y Vigilancia del puerto de Arica a inicios de 1887. En esos años, la ciudad enfrentó profundos cambios históricos. El terremoto y tsunami de 1868, la incorporación de estas regiones por la Guerra del Pacífico (1879-1883) y los primeros años de la administración chilena en la zona fueron eventos que desencadenaron modificaciones políticas, científicas y culturales. En ese contexto, a finales de 1886, emergió el brote epidémico de cólera que azotó con efectos devastadores a los países del continente y originó un quiebre en la vida cotidiana. Por su rápida difusión en la zona central y sur del país, se anticipaba su propagación a lo largo de todo el territorio, incluyendo las localidades del norte chileno.

Esta investigación aborda los problemas sanitarios relacionados con la irrupción del cólera en el puerto de Arica. El análisis de este brote en la zona demuestra las gestiones que desplegaron las autoridades y los médicos en sitios alejados del centro capital del país. A partir de ese marco se propone, desde una perspectiva local, dos escenarios de la conformación del control sanitario a escala nacional. En primer lugar, se examina la activación de una estructura institucional con el propósito de impedir el contagio de la afección. En segundo lugar, se aborda la implementación de mecanismos para aplicar los preceptos de higiene, siendo fundamentales para evitar la irrupción de la enfermedad y su afectación en los sectores más pobres de la sociedad (Rosenberg, 1966).

El brote de cólera se desarrolló en un período de cambios en los conocimientos científicos debido a los progresos de la bacteriología. Estos avances médicos se articularon en procesos más amplios relacionados con discusiones, controversias y argumentos antes de obtener una aceptación generalizada en la comunidad. A mediados del siglo XIX, los trabajos de Joseph Lister sobre el contagio por instrumentos quirúrgicos aportaron evidencias fundamentales de las formas de propagación de las enfermedades infecciosas. Estos adelantos científicos tardaron varias décadas para transitar desde el laboratorio hasta la sociedad, con mensajes que incluyeron reformas en la salud pública y las mujeres que impulsaron los cambios en el mundo doméstico (Tomes, 1999). Así, la teoría de los gérmenes atravesó etapas de gestación hasta derribar al paradigma miasmático que tenía una vigencia de varios siglos.

El cólera en el ámbito sanitario y como problema de salud pública ha preocupado a los historiadores latinoamericanos, quienes han analizado las representaciones de las epidemias que atacaron a los principales puertos del continente (Fiquepron, 2018). La situación Argentina ha sido examinada desde el disciplinamiento estatal impuesto para mejorar las condiciones sanitarias de los centros urbanos, las que se enfocaron en el control de los sectores populares y sus espacios (Aguerregaray, 2019). Además, los trabajos han abordado la definición de las infecciones como fenómeno social y la construcción de percepciones de la enfermedad, desde el punto de vista de la prensa (Pascual, 2017).

La indagación sobre la epidemia de cólera en los países de la región a mediados del siglo XIX lleva a Carbonetti (2016) a interrogarse por las consecuencias institucionales y políticas de la enfermedad. Para ello compara los brotes de 1867-1868 y 1886-1887. En el primero, los médicos debido a su escasa injerencia en la burocracia estatal, tuvieron bastantes conflictos con otros actores locales durante la gestión de la emergencia sanitaria. En cambio, en el segundo, se produjo una modernización del aparato estatal, lo que impactó en la creación de instituciones destinadas al cuidado de la salud pública y la consolidación de la élite médica. Asimismo, se ha investigado la ciudad de Buenos Aires como localidad portuaria que mantenía profundos vínculos comerciales con Europa Occidental, donde los barcos eran el principal medio para la movilización de la epidemia (Álvarez, 2012).

Los historiadores han investigado el papel del Estado, los médicos y la formación de una institucionalidad destinada a controlar las epidemias en las urbes chilenas (Muñoz, 2018; Laval, 2003). En el caso del norte, por lo reciente de la Guerra del Pacífico, el Estado de Chile se encontraba en proceso de instalación en los territorios anexados; entonces, agentes intermedios fueron los que dieron respuesta al brote epidémico (Dimas, 2020). Como plantea Savala (2022), el océano Pacífico conectó a Chile y Perú a través de flujos poblacionales, mercancías e insumos entre sus principales puertos. En ese contexto, la epidemia de cólera tuvo como medio de propagación la vía de comunicación marítima. A pesar del quiebre que provocó el conflicto bélico, ambos países formaron instancias de intercambios entre profesionales médicos, diplomáticos y actores que enfrentaron la epidemia en las dos naciones. En otras palabras, la perspectiva transnacional destaca la colaboración y el desarrollo científico-médico por sobre los antagonismos nacionales.

Tras la Guerra del Pacífico, el extremo norte de Chile experimentó la reorganización de su territorio y población, mediante el Tratado de Ancón, firmado con Perú el 20 de octubre de 1883. En su artículo tercero, estipulaba que las provincias de Arica y Tacna continuaban en posesión de Chile por un plazo de diez años, discerniéndose su dominio y soberanía vía plebiscito, acuerdo que no se concretó. La ley del 31 de octubre de 1884 organizó la división administrativa de estos territorios, otorgándole la denominación de Provincia de Tacna, la que comprendía los Departamentos de Tacna y Arica.

En la actualidad, la importancia que adquiere el tratamiento de las enfermedades en lugares lejanos de los centros políticos motiva la construcción histórica de un relato que problematice el fenómeno, sus impactos en la sociedad y los cambios que impulsó en la estructura sanitaria. En esa línea, la propuesta indaga el despliegue local de un proceso de la administración nacional. La ciudad de Arica se transformó en un espacio particular para estudiar los efectos de esta epidemia, por su ubicación geográfica y la situación post bélica en que se encontraba la zona. Además, la revisión bibliográfica demuestra la ausencia de trabajos que profundicen sobre este brote de cólera en el puerto. Una de las contribuciones de este análisis consiste en llenar este vacío y fomentar un debate entre la historia de las enfermedades y la sociedad de finales del siglo XIX.

**Figura 1:**
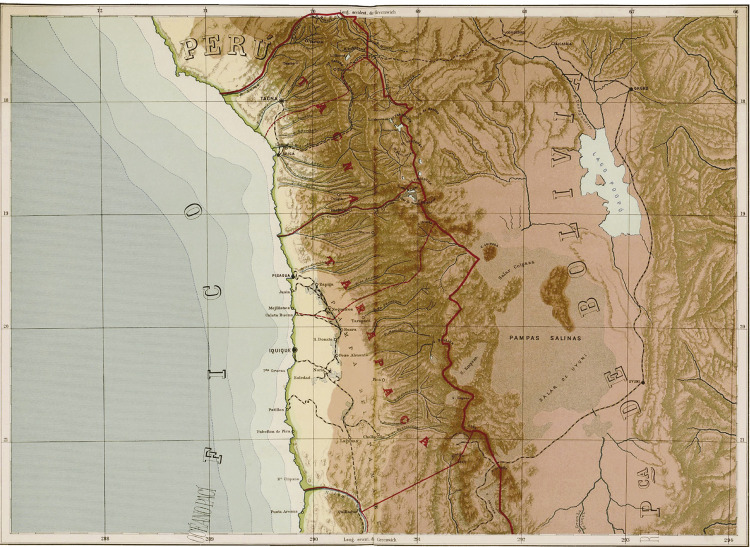
: Mapa que localiza el puerto de Arica (Chile, 1911)

Investigar el desarrollo de este brote de cólera en Arica se justifica por cuatro razones. En primer lugar, se reflexiona sobre los efectos de la epidemia en una zona portuaria fronteriza y que activó disposiciones que tenían como objetivo resguardar la salud de la población y, paralelamente, proteger el territorio de la entrada de la enfermedad. En segundo lugar, se indagan las resoluciones que las autoridades locales impulsaron para cambiar las prácticas de higiene en la comunidad ariqueña. En tercer lugar, el estudio proporciona una panorámica acerca de los conocimientos médicos de la afección y los tratamientos propuestos para abordar el malestar. En esa línea, las enfermedades y las terapéuticas para su curación se encuentran cargadas de significados que exceden lo meramente patológico (Armus, 2007). En cuarto lugar, se examinan las consecuencias de la epidemia enmarcadas en los años iniciales de la administración del Estado chileno en la región.

La investigación se encuentra sustentada por un cuerpo documental constituido por publicaciones, especialmente de médicos que describieron las características del brote epidémico, la sintomatología y las formas de contagio. También se incluye la revisión de *El Porvenir*, único periódico que se imprimía durante esos años en Arica, el que sirve como indicador relevante para establecer las trayectorias de la enfermedad en la zona y manifestó las problemáticas sanitarias, así como las medidas que tomaron los principales actores del puerto. Además, se incorpora documentación proveniente del Archivo Histórico Vicente Dagnino, registros que profundizan en las decisiones que adoptó la gobernación y entregan una panorámica de las condiciones de salubridad en la ciudad.

## La coyuntura y propagación de la epidemia

La medicina afirmaba que “el cólera es jenerado por una sustancia específica, proveniente únicamente de la India i especialmente del Alto del Ganjes” (Manterola, 1888, p.3). Se indicaba que los cuatro grandes brotes, 1830, 1846, 1865 y 1883, habían comenzado en la India y se propagaron a través de viajeros por tierra o mar. Estas sucesivas oleadas, ya fueran esporádicas, endémicas o epidémicas a lo largo del siglo XIX, se debilitaron y reaparecieron, teniendo siempre el mismo punto de origen en ciudades ubicadas en el valle del Ganges.

Las epidemias, entre esas el cólera, pueden ser consideradas como un quiebre en la sociedad. En sus inicios, la presencia de la enfermedad generó hechos triviales y que eran poco advertidos por los contemporáneos, provocando negación o indiferencia. A nivel público, solo se aceptaba la declaración de una epidemia cuando su existencia resultaba innegable. Según Charles Rosenberg (1989), su desarrollo tiene una forma dramatúrgica, tras un abrumador inicio, avanza en un escenario temporal y geográfico definido, sigue una línea argumental de creciente tensión, desemboca en una crisis y, posteriormente, sus efectos se van mitigando de manera gradual.

El brote de cólera de la década de 1860 marcó el primer impacto devastador de la enfermedad en el continente americano, afectando ciudades como Río de Janeiro, Montevideo, Buenos Aires, Rosario, Córdoba y Mendoza. Desde entonces se mantuvieron casos aislados hasta los años 1880, cuando se declaró la epidemia en varias urbes de la República Argentina. Por las muertes y los resultados que provocaba, el terror que suscitaba el cólera en las sociedades estuvo latente. Apenas se manifestaban los primeros afectados, los habitantes abandonaban rápidamente las ciudades, “salen todos precipitadamente y así se ve más cólera en Mendoza, Rosario, Córdoba, con el ferrocarril, donde con más facilidad llegan” (Cannon, 1887, p.6).

En las provincias argentinas, el aumento exponencial de víctimas tuvo un efecto demográfico significativo. Cabe señalar que el Estado presentaba dificultades para cuantificar el movimiento de la población debido a las complicaciones de comunicaciones entre las instituciones y la falta de homogeneidad en los procedimientos para recolectar cifras. Según Hamlin (2009), la política juega un papel importante en la formación del gobierno sanitario y articula los consensos necesarios para la activación de medidas. A las consecuencias sanitarias de la epidemia, se agregaban trastornos económicos y sociales. En los tiempos más álgidos del brote, el médico y político Benjamín Araoz (1887, p.5) describía que “el comercio cerraba sus puertas, la miseria se dejaba sentir en la gente que quedaba sin trabajo, y la duda, el dolor, el espanto parecían doblegar a aquel pueblo sobre el cual soplaba recio un hálito de muerte”.

A medida que el brote se expandía rápidamente por el territorio argentino y alcanzaba la zona cordillerana, el gobierno de Chile adoptó normas especiales para evitar la llegada de la epidemia (Puga Borne, Lastarria, 1887, p.III). Dada la súbita irrupción del mal, se requería tomar precauciones con anticipación. En diciembre de 1886, fue creada una Comisión de Higiene Pública responsable de estudiar, diagnosticar y establecer las órdenes pertinentes para combatir el brote epidémico. El 3 de enero de 1887, esta organización fue reemplazada por la Junta General de Salubridad, manteniendo atribuciones similares a la anterior. Su funcionamiento duró solo unos meses. En noviembre, se conformó la Comisión Directiva del Servicio Sanitario del Cólera, con el objetivo de manejar el brote epidémico. La formación de estas tres instituciones, en menos de un año, para enfrentar la grave amenaza del cólera refleja una etapa de construcción de la burocracia sanitaria. En Chile, previo a la apertura del Consejo Superior de Higiene en 1892, coexistían varios organismos encargados de la administración sanitaria, entre ellos las Juntas de Beneficencia, la Facultad de Medicina, las municipalidades y las gobernaciones.

La situación de Argentina obligó a las autoridades chilenas a instalar cordones sanitarios en la Cordillera de los Andes, vigilados por fuerzas militares. El presidente de Chile, José Manuel Balmaceda, decretó la prohibición “hasta nueva orden de toda comunicación con la República Arjentina por la cordillera de los Andes. Solo la correspondencia será recibida en los resguardos e introducida con las medidas de precaución dictadas por el Ministro de Interior” (El Porvenir, 1886-1887, [20 ene. 1887, p.2]). Sin embargo, en la práctica, los cordones sanitarios demostraron ineficacia porque no lograron contener la transmisión del cólera. Para el médico Mamerto Cádiz (1917, p.8), la profilaxis internacional, “no sirve porque los viajeros burlan los cordones por caminos estraviados para escapar a las pérdidas de tiempo que les imponen las cuarentenas”. A su vez, por el tiempo que demoraba la incubación del mal, una persona podía salir desde Argentina en perfecto estado de salud y llegar a Chile con los primeros síntomas (Herrera, 1887, p.17).

Los brotes epidémicos permitieron la formación de políticas públicas sanitarias profilácticas para evitar la difusión del contagio (San Martín, 2016). Sin embargo, la aplicación de estas medidas estuvo envuelta en discusiones políticas, económicas y geográficas. En ese marco, se intentaba fortalecer la prevención a través de los controles y la revisión del tránsito a nivel fronterizo. Se consideraba que el aislamiento era una de las herramientas más efectivas para impedir la difusión del flagelo en el país. Según la Facultad de Medicina, la principal acción precautoria era la organización de un servicio de sanidad, ya que los medios de transportes facilitaban los contactos, “acortan las distancias i aproxima los países” (Murillo, 1886, p.4). A ello se agregaban, los avances de la revolución industrial que se expresaron en la rapidez que alcanzaron los medios de transportes y el fortalecimiento del comercio internacional (Huber, 2006).

El brote de cólera que azotó a Chile, entre finales de 1886 y 1888, causó alarma en el país por su rápida expansión y elevada mortalidad. El mal colérico atacó la provincia de Aconcagua, especialmente a la ciudad de Santa Rosa, en las cercanías de Santiago. En enero de 1887, se registraron los primeros casos de diarrea simple en la población. Se creía que la naturaleza benigna de la enfermedad se debía a las altas temperaturas veraniegas y al consumo de frutas verdes. La comunidad santarrosina “jamás se dio cuenta de la gravedad del cólera, i por tanto no estuvo poseída de ese pánico propio de las personas que viven en las grandes ciudades” (Mesa, 1887, p.23). En cuanto se manifestaba la epidemia, la mayoría de los habitantes se dispersaba hacia zonas aledañas, principalmente a los campos.

En esa época, los puertos constituían una de las principales vías de ingreso y contacto del país con los mercados exteriores. Ahí, el transporte se convertía en el medio de transmisión del cólera a larga distancia, llevándolo desde lugares contaminados a otros indemnes. En ese contexto, se produjo una alteración en el comercio y las comunicaciones. Respecto a la conexión marítima, el gobierno decretó la prohibición de ingreso a los vapores venidos de Montevideo y todos los puertos argentinos. Ante el cierre, la Gobernación Marítima de Arica instruyó que los buques impedidos para continuar su ruta por la falta de agua o víveres podrían ser abastecidos de los artículos por una embarcación que debía ser destruida una vez que cumplía con su función (El Porvenir, 1886-1887, [18 dic. 1886]). Posteriormente, la Junta de Sanidad Marítima de Arica, para resguardar la salubridad del lugar, acordó “no permitir el desembarque de ningún pasajero, procedente de alguno de los puertos situados al sur de Antofagasta sin que previamente haya tomado a bordo un baño de desinfección y desinfestado su equipaje” (El Porvenir, 1886-1887, [20 ene. 1887, p.2]).

A mediados del siglo XIX, surgieron dos teorías para ilustrar el origen del cólera. En primer término, la miasmática postulaba que la afección era provocada por el mismo agente pestilencial responsable del tifus, sarampión y fiebre amarilla. Este componente “viviría o se conservaría en el suelo, en las aguas i podría ser trasportado a distancia por el aire i las corrientes humanas” (Ugarte, 1887, p.4). En segundo término, emergió el paradigma bacteriológico, respaldado por los descubrimientos de Robert Koch en Alemania y Louis Pasteur en Francia, cuyos trabajos generaron transformaciones en la etiología y terapéutica de varias enfermedades. La coexistencia de ambas perspectivas no logró consenso en el gremio médico; tuvieron seguidores y detractores que discutieron las aplicaciones de estas propuestas en el campo de la higiene (Latorre, 1888, p.28; Salazar, Newman, 1888).

En esa época, se originó un debate sobre la forma de propagación de la enfermedad (Ackernecht, 1948). En primer lugar, los contagionistas señalaban que la difusión de la afección podía darse entre personas mediante el contacto físico, la utilización de objetos contaminados o la respiración. En segundo lugar, los anticontagionistas indicaban que la transmisión se explicaba por el efecto de los miasmas, los que provocaban una putrefacción de la atmósfera y cuya respiración inoculaba la bacteria en las personas (Carbonetti, 2003). Estas propuestas demostraron las relaciones entre la ciencia y política, ya que los avances en los conocimientos se expresaron en los métodos ocupados para defenderse de las pestes como las cuarentenas y los cordones sanitarios o, simplemente, eliminar las restricciones a los viajes porque habían fracasado para detener el contagio de la peste (Huber, 2006).

Según la bacteriología, el origen del cólera residía en el *bacilus coma*, que se alojaba en los organismos de animales y plantas, multiplicándose rápidamente cuando se encontraba en el intestino humano. La identificación de los gérmenes como el factor causante del cólera proporcionó herramientas para la profilaxis general y frenar la extensión de la epidemia. En ese instante, el trabajo científico fue clave para definir los mecanismos de transmisión o los sitios donde se albergaba la bacteria. La discusión se centraba en los vehículos de propagación; por un lado, los especialistas sostenían que los enfermos eran los difusores, mientras que otros consideraban las mercaderías, las corrientes atmosféricas y los objetos. De igual manera, se requería determinar que las aguas eran un medio de infección, por encima de otros como los terrenos o las deposiciones de los contagiados. La ciencia también debía precisar el tiempo de incubación, los tratamientos más efectivos y qué órganos del cuerpo humano se veían más afectados por la enfermedad (Koch, 1887).

El cólera corresponde a una enfermedad de tipo gastrointestinal. Para los especialistas, los principales síntomas del cólera eran “vómitos i diarrea, enfriamiento general, sudor frío i viscoso, calambres, disminución o supresión de la orina, pérdida de la voz, cara azulada i postración profunda del ánimo i de las fuerzas” (Díaz, 1887, p.3). Sin embargo, la etiología de la enfermedad reconocía múltiples factores como detonantes de la afección. En ocasiones, los especialistas afirmaban que “aparece el cólera de noche i en compañía de un resfriado; deben por tanto evitarse todas las causas de estos últimos: falta de abrigo, cambios de ropas imprudentes, bebidas frías en gran cantidad, uso de helados” (Comisión de Hijiene Pública de Chile, 1887, p.5). El fallecimiento sobrevenía a las pocas horas, cuando se manifestaba de manera grave.

Para los epidemiólogos, el origen y la transmisión del cólera se explicaba por dos factores. En primer lugar, el agente infeccioso y las características físicas del enfermo, como la historia clínica, la nutrición y la edad. En esta época, se observaba la existencia de perfiles predispuestos a contraer el cólera, “las personas propensas a padecer diarrea, así como las que sufren otras enfermedades. Los borrachos por costumbre y los individuos que llevan una vida relajada” (Biblioteca Homeopática Doméstica, 1909, p.6). En segundo lugar, se vinculaba con el ambiente en que se encontraba la población, lo que incidía en los medios de propagación de la afección. Se indicaba que “el desaseo es favorable a su desarrollo, i que hace sus primeras víctimas entre los individuos debilitados por la miseria, por los vicios i por enfermedades anteriores” (De la Barra, 1887, p.6).

Uno de los puntos más debatidos a nivel de los especialistas era comprender las vías de contagio del cólera. Según el médico Puga Borne (1886, p.5), bacteriológicamente, el cólera era “infecto-contajioso en el sentido de trasmitirse al hombre sano por la acción de ciertos productos del enfermo”. En la literatura, se detallaban episodios de la difusión infecciosa por el contacto entre personas, lo que permitía formar una trayectoria de la enfermedad. La frecuencia de casos y la descripción de la sintomatología destacaban las heces entre las principales fuentes de infestación, “el colérico, al depositar sus excrementos, deja en la letrina un foco poderosísimo de infección que contamina a los que después hacen uso de ella” (Lira Errázuriz, 1886, p.7). Por ese motivo, el grupo familiar y comunitario se encontraba en riesgo de contraer la epidemia. En este recorrido, cada individuo era portador del mal y podía transportarlo de un lugar a otro.

El tratamiento médico del cólera, en ocasiones resultaba poco efectivo por el rápido progreso del cuadro en el enfermo. La gravedad de la enfermedad se exacerbaba con el aumento de la deshidratación, y el tratamiento médico oportuno permitía disminuir la tasa de mortalidad. A pesar de los avances en los conocimientos científicos, existían variados métodos de curación para tratar el mal, los que dependían de la edad, constitución o estado del afectado. Entre la amplia terapéutica, la aplicación de inyecciones o medicamentos solo podían contener la deshidratación acelerada de la persona (Aguirre, 1887a). Por ese motivo, la legitimación de los procedimientos terapéuticos entre la comunidad médica y el trabajo en el laboratorio eran importantes para “dejar constancia de los resultados obtenidos en el lazareto por los tratamientos puestos en práctica” (Aguirre, 1887b, p.23). Con esta base, se lograba entender las características del cólera, sus complicaciones frecuentes y las fórmulas más efectivas para mitigar sus efectos.

## El gobierno local y las fases del cólera

La gobernación de Arica era la encargada de la administración y el gobierno local, entre ellas las funciones del ámbito sanitario. Ante la inminencia del arribo de la epidemia, a fines de 1886, delegó sus facultades en el comité de higiene y vigilancia pública del puerto. La dirigencia civil determinó que los esfuerzos debían concentrarse en impedir que el flagelo del *cólera morbus* llegara al departamento. Ante esta emergencia, se planteaba que “las medidas que puede adoptar la autoridad, carecerían de eficacia sin el concurso de las personas influyentes e ilustradas que llevando el consejo i la persuasión al pueblo, logren aunar todas las voluntades” (Archivo Histórico Vicente Dagnino, 1886-1887, v.290, [21 dic. 1886]). Así, para la formación del comité de higiene, se convocó a miembros representativos de la comunidad ariqueña que constituían parte de la élite local. Entre ellos, se encontraban los médicos Tomás Aravena, Eduardo Rodríguez Prieto, Pedro A. Ramírez y Mariano Martínez, el presbítero Saturnino Bernal, el coronel Manuel Novoa y los vecinos Federico Soza, Pablo Ramos, Carlos Nugent, Napoleón Casey, Domingo Pescetto, Pastor Matienzo, Blas Tagle, Emilio Belcke, Jénaro Cáses, Guillermo Finlayson, Alejandro Visscher y Manuel Núñez.

La instancia gestionó los medios para proteger a la población de la epidemia de cólera y discutió la organización para abordar su potencial arribo a la localidad. Sus acciones tenían como objetivo el control de la higiene de la urbe y vigilar el cumplimiento de las normas. Una de las primeras disposiciones fue “dividir la población en tres cuarteles y nombrar seis miembros de la Junta para cada uno de ellos” (El Porvenir, 1886-1887, [28 dic. 1886, p.2]). La finalidad de esta distribución era estudiar las decisiones más atingentes, según el sector del radio urbano. En la práctica, los dirigentes vecinales recorrieron los barrios e indagaban sobre las necesidades del lugar. Paralelamente, la municipalidad formuló ordenanzas para llevar a cabo la inspección de viviendas, fiscalización de mercados, prohibición de mantener animales en el radio urbano y la limpieza de las calles.

Desde el Comité de Higiene y Vigilancia, el trabajo comunal era fundamental para indagar las características de la salubridad en la ciudad y la distribución en cuarteles que eran responsables de presentar comunicaciones al organismo. Para cada uno de ellos fue nombrado un médico encargado de redactar contenidos técnicos a la autoridad. El primer cuartel, localizado en el sector costero, solicitaba a los fleteros el aseo de la playa, la limpieza del muelle, extraer el guano y permitir la matanza de los lobos marinos de la Isla Alacrán. Respecto al segundo cuartel, ubicado en la zona céntrica, proyectaba la aniquilación de las jaurías de perros, prohibir la venta de carnes que tuvieran más de 24 horas y suprimir los animales al interior de las viviendas, ya que “los cerdos y demás cuadrúpedos son propensos al desarrollo epidémico, y aún las aves, habrá que especificar la cantidad que deben conservar” **(**El Porvenir, 1886-1887, [1 ene. 1887, p.2]). En cuanto al tercer cuartel, denominado “Lumbanga”, informaba que las habitaciones estaban desaseadas y sobre la presencia de focos infecciosos por la gran cuantía de desperdicios.

En esa época, extensos sectores de la población se encontraban sumidos en la pobreza, viviendo en circunstancias de salubridad precarias. Para los médicos, “el cólera ha sido la epidemia de la jente pobre e indolente que no ha desinfectado sus casas i que ha llegado a arrojar las deyecciones de los enfermos en el suelo mismo de la pieza o del patio vecino” (Koch, 1887, p.65). En esa línea, se destacaban los espacios con malas condiciones higiénicas y la falta de aplicación de estrategias preventivas. A ello se agregaba, que los individuos “no pueden dedicar mucho tiempo a los cuidados domésticos, porque tienen que atender al primero de todos, que es su subsistencia y la de sus hijos, y menos pueden gastar en desinfectantes” (El Porvenir, 1886-1887, [16 dic. 1886, p.2]).

En este contexto, resaltaba la acción filantrópica de las autoridades y los vecinos que se ocupaban de la beneficencia comunitaria. Según los juicios de la prensa, los habitantes pobres no podrían cumplir con las normas de higiene, tales como realizar baños diarios, usar desinfectantes y tener una alimentación adecuada. Por esta razón, se dispuso la distribución gratuita de este tipo de artículos. La gobernación identificó que en Caleta Vítor, ubicada a unos sesenta kilómetros de Arica, existían reservas de sulfatos de cobre y hierro, elementos utilizados como desinfectantes. Por ese motivo, se autorizó “a Demetrio Ponce, con el bote Nº 24 para atracar i cargar en ella las enunciadas sustancias i conducirlas a este puerto a consignación de esta gobernación” (Archivo Histórico Vicente Dagnino, 1886-1887, v.290, [4 ene. 1887]).

Posteriormente, se promulgó la Ordenanza General de Salubridad en Santiago, con aplicación en todo el territorio nacional. Esta normativa organizó las Juntas Departamentales de Salubridad, dotadas de facultad para dividir cada departamento en secciones, facilitando la ejecución de medidas. La Junta Departamental de Salubridad de Arica compuesta por el alcalde, médico, cura párroco, miembros de la beneficencia y vecinos, mayoritariamente conformada por integrantes del anterior Comité de Higiene y Vigilancia (Archivo Histórico Vicente Dagnino, 1887-1888, v.291, [11 feb. 1887]). Una de sus primeras decisiones fue intensificar la supervisión mediante las visitas domiciliarias, manteniendo la división de la ciudad en tres cuarteles.

La potencial irrupción del cólera afectó el comercio de la ciudad. La actividad mercantil de la provincia, tanto de Arica como Tacna, dependía de sus relaciones con los valles peruanos y Bolivia. La suspensión de las comunicaciones se vislumbraba como un escenario más perjudicial que el cólera debido a las difíciles condiciones económicas. Dada la magnitud y rápida propagación de la epidemia, se consideró la alternativa de clausurar el puerto de Arica. Teniendo en cuenta que Bolivia instaló un cordón sanitario a lo largo de sus fronteras del norte y el oeste, afectando el tráfico ariqueño. Siguiendo una medida similar, el gobierno peruano también dispuso restricciones. Sin embargo, la administración chilena decidió evitar la declaración de cierre de los puertos, ya que esto implicaría reconocer oficialmente la presencia de la epidemia e informaría a nivel internacional que estas regiones se encontraban infestadas. A juicio de la autoridad, el empleo de este régimen de excepción produciría efectos negativos en el movimiento comercial de la zona (El Porvenir, 1886-1887, [5 mar. 1887]).

La actuación de los médicos durante el brote epidémico fue una experiencia que contribuyó al avance de los conocimientos científicos de las enfermedades y, al mismo tiempo, influyó en la formulación de normativas para evitar el contagio. El progreso en el ámbito bacteriológico permitió determinar los mecanismos de difusión de las enfermedades y demostrar la efectividad de algunas medidas por sobre otras. Partiendo de esta base, se establecieron medidas de profilaxis general que impactaban a nivel comunitario y las de carácter individual que cada persona podía aplicar en su hogar. En este período de apertura comercial fue necesaria la burocratización de los movimientos y la regulación del tránsito fronterizo (Huber, 2013). Por ejemplo, se constató la eficacia de fortalecer las aduanas mediante exhaustivas revisiones médicas, en contraste con los cordones sanitarios que impactaban fuertemente en los intercambios comerciales.

A lo largo de la historia, las epidemias han afectado los movimientos comerciales, generando escasez de productos, falta de mano de obra y alza en los valores a nivel mundial. En los últimos meses de 1886, las informaciones oficiales oscilaron entre casos probables de cólera en la ciudad y la adopción de enfoques preventivos frente a la epidemia. En este contexto amenazante para el comercio y la salud pública, “los especuladores han principiado a elevar el precio de los víveres y de los artículos de primera necesidad, y a afligir con la carestía a todo el mundo y más que a nadie a los pobres” (El Porvenir, 1886-1887, [15 ene. 1887, p.2]). El aumento de los costos, principalmente de los productos básicos, se realizó a pesar de que el puerto seguía operando con normalidad. Este encarecimiento podría haberse explicado solo por un cierre prolongado que causase desabastecimiento de los comestibles.

El brote de cólera produjo cambios en los hábitos alimenticios de la población. Los médicos sugirieron el consumo de agua hervida y productos cocidos para eliminar los gérmenes. Para la bacteriología, estaba comprobado que las personas que ingerían agua cocida y evitaban las frutas o verduras crudas tenían menores posibilidades de contraer el malestar. Esta precaución era enfatizada en el caso de aguas cuyo origen era desconocido, ya que tenían mayores probabilidades de contaminación (Fonck, 1887 **,** p.13). Según definieron los especialistas, “el calor aplicado convenientemente a las bebidas i alimentos destruye con certeza todo jérmen de cólera” (Bourgeois, 1888, p.9). Entre otras recomendaciones, se hacía hincapié en la limpieza de utensilios de mesa, manos y cualquier otro elemento cercano a los comestibles.

En esta época, el control municipal de los alimentos y bebidas era importante para evitar el comercio de productos nocivos y que podían transformarse en focos de la enfermedad colérica. Por consiguiente, se prohibió arrojar desperdicios de comestibles en lugares públicos, expender bebidas o licores adulterados y “vender frutas verdes o en descomposición, carnes en mal estado o de mala calidad, pescado descompuesto o muerto con dinamita” (El Porvenir, 1886-1887, [4 ene. 1887, p.2]). Con la puesta en marcha de esta fiscalización, se denunciaba “la mala calidad de las bebidas que se expenden en ciertos establecimientos, al extremo de haber sido frecuentes los ataques de enfermedad al estómago” (El Porvenir, 1886-1887, [14 dic. 1886, p.2]). En Arica, se reforzó la inspección de alimentos con el propósito de evitar la propagación desde las zonas afectadas. Por esta razón, se prohibió “la introducción i venta en la ciudad de sandías i peras en prevención de las funestas consecuencias que trae el uso de estas frutas, bajo las penas de comiso i demás prescritas en las leyes” (Archivo Histórico Vicente Dagnino, 1886-1887, v.290, [16 feb. 1887]).

Hasta fines del siglo XIX, la ciudad no contaba con una institución encargada de la inspección de los alimentos comercializados, y el mercado no tenía un perito químico o médico para determinar las características de los productos mediante análisis. Con el propósito de resguardar la salud pública y llevar a cabo el control de los licores que se comerciaban, la Junta de Salubridad designó un inspector de líquidos (Archivo Histórico Vicente Dagnino, 1886-1887, v.290, [26 feb. 1887]). Tiempo más tarde, esta elección fue anulada por la falta de fondos municipales destinados para su contratación (Archivo Histórico Vicente Dagnino, 1886-1887, v.290, [2 mayo 1887]). Posteriormente, fue nombrada “una comisión ensayadora, compuesta del facultativo doctor Eduardo Rodríguez i del farmacéutico Belisario Palacios, para que procediendo de acuerdo, analice los licores que la Gobernación someta a su examen” (Archivo Histórico Vicente Dagnino, 1886-1887, v.290, [14 mar. 1887]). Ante la emergencia epidémica, se produjo la construcción de una burocracia municipal ocupada de vigilar la calidad de los productos alimenticios. Así, varias instituciones y secciones del gobierno local surgieron como respuesta a necesidades indiscutibles y cotidianas.

Los médicos promovieron iniciativas para difundir los conocimientos del cólera entre la comunidad, tanto los síntomas como los posibles remedios (Navarrete, 1887; De Grand-Boulogne, 1887). Las autoridades planificaron medidas enfocadas en la prevención y la higiene, más allá de los procedimientos coercitivos que significaban los cordones sanitarios o las cuarentenas. La *Cartilla Sanitaria del Cólera* fue una publicación de instrucciones dirigidas a la población, dando a conocer los mecanismos de transmisión, algunos métodos de curación y describía las consecuencias del flagelo en otras naciones. En este trabajo, se proporcionaban consejos para prevenir el contagio de la enfermedad. La propuesta reforzaba una concientización frente a la afección, divulgando saberes referentes con el aseo personal, la higiene domiciliaria y la adopción de un régimen de alimentación adecuado (Fernández, 1886). Siguiendo estas directrices, la gobernación se preocupó por distribuir cien ejemplares de las Instrucciones sobre el *Cólera y de las Cartillas Higiénicas*, ambas aprobadas por la Comisión de Higiene Pública (Archivo Histórico Vicente Dagnino, 1887-1888, v.291, [30 mar. 1887]).

## La higiene en la ciudad

Hasta mediados del siglo XIX, el suministro de agua en el puerto de Arica provenía de arroyos ubicados en la parte alta del terreno, los cuales desembocaban en el mar y se alimentaban por filtraciones del río San José. Sin embargo, debido al crecimiento del radio urbano y su emplazamiento en una zona permeable con napas subterráneas, se tuvo que recurrir a la excavación de pozos para obtener agua potable. Según un informe de la Dirección de Obras Públicas, “el agua de pozos es la única potable de que actualmente se surte esta población, i ésta es mala por recibir en la parte baja las filtraciones” (Archivo Histórico Vicente Dagnino, 1887-1888, v.291, [24 abr. 1888]). Este problema se agravaba cuando las materias excrementicias se mezclaban con el líquido que se ingería, ya que “los pozos colocados a poca distancia de las letrinas no quedan exentos de la permeabilidad del suelo” (El Porvenir, 1886-1887, [11 ene. 1887, p.2]). El agua constituía el ambiente propicio para la bacteria del cólera y, al mismo tiempo, era su medio conductor primordial. Por esta razón, los gobiernos planificaron mejoras a largo plazo, materializadas en obras de saneamiento como la instalación de cloacas para verter los contenidos de los desagües en el mar y la construcción de acueductos que permitieran separar los desechos del agua destinada al consumo.

En Arica, como en la mayoría de los centros urbanos, la provisión de agua potable mediante cañerías era bastante escasa. Se distribuía a través de pozos y aguadores, quienes circulaban por las calles vendiéndola. Existían pozos públicos en la parte alta de la ciudad y “la fuente del puerto viejo de donde se surten un gran número de pobladores” (El Porvenir, 1886-1887, [27 ene. 1887, p.2]). Los médicos consideraban que el uso de este vital recurso era uno de los principales desencadenantes de la afección, asegurando que la trayectoria de los casos epidémicos seguía los cursos de agua. La corriente recogía los desechos presentes en los conductos o era contaminada por los pozos que funcionaban como letrinas. Ese líquido, conducido a través de la urbe, se ocupaba para la bebida o lavar ropas, lo que aumentaba las posibilidades de contagio. Como medida preventiva, se indicaban los peligros de su consumo en las localidades atacadas por el cólera, recomendando hervirla, almacenarla en botellas y “sacudirla repetidas veces para hacerla reconquistar el aire que ha perdido a causa de la ebullición” (Castañé, 1886, p.16).

En esa época, el sistema de acequias era el principal método que se ocupaba en la ciudad para la distribución de agua o la evacuación de excretas domiciliarias. La autoridad local, preocupada por la higiene pública, dispuso la limpieza de las acequias, estableciendo un “servicio organizado que permita arrojar el cieno directamente a los vehículos que hayan de trasportarlos a los lugares elejidos y arrojarlo en zanjas que deberán ser cubiertas inmediatamente” (El Porvenir, 1886-1887, [7 dic. 1886, p.2]). Esto se complementó con una proporción abundante de agua en las acequias que realizaban el servicio público y su limpieza. Además, se destinaron carretones para sacar las aguas sucias de la ciudad, las que eran trasladadas a pozos alejados de la comunidad, en conformidad con la prohibición “de arrojar aguas sucias o inmundicias en las calles, plazas i lugares públicos, o en sitios no cerrados de la población” (Archivo Histórico Vicente Dagnino, 1886-1887, v.290, [31 dic. 1886]).

La gobernación de Arica adoptó medidas de higiene para mejorar la salubridad pública y evitar la irrupción de la epidemia. En ese contexto, se distinguieron dos tipos de prácticas: en primer lugar, las reglas de higiene pública, establecidas por las administraciones nacionales o locales; en segundo lugar, las prevenciones de higiene privada que abarcaban los hábitos individuales para evitar el contagio (Comisión de Hijiene Pública de Chile, 1887, p.3). Se adoptaron normativas de higiene que delimitaron las acciones de la autoridad local en la ciudad, incluyendo labores como la quema de los desechos, el depósito de basuras y la limpieza de las calles. En ese marco, la gobernación ordenó el barrido de calles y caminos públicos al menos tres veces a la semana (Archivo Histórico Vicente Dagnino, 1886-1887, v.290, [3 feb. 1887]). Se proporcionaban pautas sobre el manejo de los desperdicios para evitar la formación de focos de infección, sugiriendo que “la extinción debe llevarse a cabo por medio de incineraciones repetidas ya sea conglomerando parcialmente ya usando la parafina para procurar la combustión” (El Porvenir, 1886-1887, [8 ene. 1887, p.2]). Con la finalidad de preservar la salubridad pública, se ordenó “el cerramiento de la gran cantidad de sitios abiertos que existen en esta población” (Archivo Histórico Vicente Dagnino, 1886-1887, v.290, [27 ene. 1886]), lo que evitaba que estas zonas se convirtieran en zonas de basurales. También se destinaron nueve carretones de la policía de aseo para la extracción de basuras, los que incorporaban una campanilla para anunciar su arribo a los distintos barrios (El Porvenir, 1886-1887, [12 mar. 1887]).

Previo al arribo del cólera, el Ministerio de Justicia recomendaba la observación de lugares con altos índices de hacinamiento, como colegios y cárceles. En cuanto a los primeros, se señalaba que “los locales en que funcionan las escuelas públicas de este departamento son insalubres” (Archivo Histórico Vicente Dagnino, 1886-1887, v.290, [11 jun. 1886]). El doctor Jacinto Ugarte, comisionado por el gobierno para inspeccionar las escuelas públicas de la provincia, informaba sobre la pésima situación de salubridad en la escuela de niñas, destacando que “carece de patios i de todas condiciones indispensables para un establecimiento de enseñanza” (Archivo Histórico Vicente Dagnino, 1887-1888, v.291, [2 jun. 1888]). Respecto a los recintos penitenciarios, el comandante de policía fue instruido para realizar “el más esmerado aseo en todos los pisos de los patios, que hará regar con algún desinfectante; en los calabozos, los que hará limpiar y desinfectar en sus pisos, techos y paredes” (El Porvenir, 1886-1887, [21 dic. 1886, p.2]). En ese momento, se pensaba que la aglomeración de individuos en lugares estrechos y “poco ventilados se desprenden emanaciones miasmáticas que son las que sentimos al entrar en la mañana en un dormitorio cerrado” (Jiménez, 1887, p.4). De esa manera, el aire era identificado como una de las principales fuentes de transmisión y contagio de enfermedades.

En los años siguientes a la Guerra del Pacífico, las regiones del norte de Chile conservaron un alto contingente militar. El cirujano primero del Regimiento n.2 de Artillería expresó a las autoridades su preocupación acerca de la posible aparición del cólera asiático, señalando que esta enfermedad “produce mayor mortalidad en aquellos parajes, en donde se encuentran acumulados mayor número de individuos” (Archivo Histórico Vicente Dagnino, 1887, v.209, [26 ene. 1887]). En esa línea, las instalaciones del Regimiento albergaban gran cantidad de personas, transformándolo en un espacio de riesgo. Para mitigar esta situación, era necesario llevar a cabo diversas acciones con el fin de mejorar la higiene del establecimiento. Estas consistían en eliminar la presencia de sustancias orgánicas en el suelo, realizar el blanqueo del lugar, sellar y desinfectar los excusados del cuartel. Además, se recomendaba a la tropa tomar medidas preventivas, como evitar resfriados, abstención de comidas abundantes, privarse del consumo de frutas y licores espirituosos. Para cumplir con estas disposiciones, el comandante de armas solicitó a la gobernación de Arica “dos carretones para el aseo del regimiento” (Archivo Histórico Vicente Dagnino, 1887, v.209, [31 ene. 1887]).

A partir del brote de cólera, se reformaron los espacios de la muerte en el radio urbano. Esto ocurrió en un período histórico en que predominaba la fundación de cementerios alejados de las ciudades. En Arica, los dos cementerios se encontraban en estado de abandono, originando peligros para la higiene y la salubridad pública. Incluso, se acusaba la presencia de “cadáveres hoi espuestos al aire libre en los nichos descubiertos i sepulturas abiertas de dichos cementerios” (Archivo Histórico Vicente Dagnino, 1886-1887, v.290, [27 ene. 1886]). Por ello, la gobernación dispuso la “demolición del antiguo panteón que se halla casi en la misma población. El mejoramiento del que actualmente sirve para las defunciones, por no hallarse en condiciones propias ni higiénicas” (El Porvenir, 1886-1887, [4 ene. 1887, p.2]). Asimismo, las autoridades procuraron que los fallecidos por cólera fueran sepultados rápidamente y que los ataúdes estuvieran sellados de modo hermético. El Regimiento n.2 de Artillería de Costa habilitó un carro mortuorio para su utilización sin gravamen.

Finalizando el verano de 1887, se afirmaba que el peligro de un brote devastador en las ciudades del norte de Chile disminuía. En esos días, la epidemia arreciaba en la región central, sobre todo en Santiago. Según la prensa, este contraste se dio por varios factores, en Arica el clima no sería propicio para el desarrollo de la afección, las medidas de las autoridades que impidieron la difusión del contagio y “la morigeración de las costumbres del pueblo, que palpando los efectos de la enfermedad, se ha sujetado a un régimen higiénico conveniente, obedeciendo a las prescripciones de la ciencia” (El Porvenir, 1886-1887, [5 feb. 1887, p.2]).

En abril de 1887, se produjo la reapertura de las fronteras de Perú y Bolivia, se informaba que la epidemia de cólera “declina rápidamente en todos los lugares atacados por ella, pudiendo considerarse como casos aislados los que se presentan” (El Porvenir, 1886-1887, [14 abr. 1887, p.2]). En términos más amplios, y en diálogo con los procesos científicos vivenciados en la región, las medidas para enfrentar la epidemia articularon, por un lado, los argumentos expuestos por los médicos y su capacidad para trabajar en conjunto con las autoridades políticas, y, por otro, conciliar la actividad comercial e industrial con las medidas destinadas a frenar el contagio.

A finales del mismo año, la prensa continuaba informando casos de defunciones diarias en Santiago y Valparaíso (El Porvenir, 1886-1887, [7 dic. 1887]). La Intendencia de Tarapacá, frente al peligro latente que significaba el cólera, acordó “prohibir la introducción a este puerto de toda clase de frutas que se traigan del sur y entre las que se traigan del norte, el plátano, la sandía y el melón; y respecto de las legumbres las que se traigan también del sur y que no se acostumbre a comer cocidas” (El Porvenir, 1886-1887, [30 dic. 1887, p.3]). En Arica, durante 1888 surgió el carbunclo en los ganados de Lluta, “enfermedad que puede considerarse epidémica, ya que ha hecho innumerables víctimas i se ha comunicado a los animales de otras haciendas vecinas” (Archivo Histórico Vicente Dagnino, 1887-1888, v.291, [14 abr. 1888]). La autoridad dictó medidas para evitar el contagio desde el ganado vacuno a los humanos.

## Consideraciones finales

El mortífero brote de cólera que azoló al centro de Chile entre 1886-1888, en Arica no generó impactos demográficos significativos. A diferencia de otras epidemias, como la fiebre amarilla que estalló en la ciudad, tras el terremoto de 1868 por la destrucción de la infraestructura sanitara o la malaria que afectó de manera endémica a la región (Soto, Chávez, Pizarro, 2019). Sin embargo, el rápido avance de la afección en el continente, las imágenes brutales de otros lugares y la proximidad del contagio en la zona norte del país condujeron a las autoridades locales a tomar varias medidas orientadas a la higiene y crearon una institucionalidad responsable para evitar el arribo del cólera. Desde la perspectiva expuesta a lo largo del artículo, se puede apreciar la conexión de las normativas y que permiten vincular el abordaje transnacional de la enfermedad.

La posible llegada del cólera al puerto de Arica generó estructuras destinadas a gestionar el ámbito sanitario. El Estado chileno se encontraba en proceso de instalación debido a la reciente anexión de estos territorios tras la guerra, lo que derivó en el fortalecimiento de instancias locales para luchar contra el cólera. Fueron varios los actores que desde el inicio participaron en la gestión de este brote. Con ese propósito y bajo su alero, se aplicaron normativas y ordenanzas que trataron de reducir el impacto de la epidemia en la comunidad, así como se activó una política que vinculó a los médicos chilenos y los peruanos.

El Comité de Higiene y Vigilancia Pública del Puerto, la Junta Departamental de Salubridad, la municipalidad y los médicos adoptaron distintos métodos para contener la aparición del mal, entre ellos leyes, normativas y visitas domiciliarias. Esta perspectiva estuvo sustentada en la higiene de los espacios públicos y en la promoción de la limpieza individual. En esta línea, el radio urbano fue dividido en tres cuarteles, donde se estudiaron las condiciones, se elaboró un diagnóstico y se propusieron normas destinadas a la desinfección de los entornos. La conformación de este andamiaje institucional se ocupó de regular el comercio alimenticio, la presencia de animales en las casas y extender la vigilancia de los reglamentos locales.

Este brote de cólera se enmarca en el período de los descubrimientos de Pasteur y la revolución que significaba el paradigma bacteriológico. Estos cambios sobre los tratamientos de la enfermedad tuvieron una transición gradual, en que predominaba la nueva óptica con los criterios de la teoría miasmática. En algunas ocasiones, las propuestas médicas supusieron una combinación de ambos principios que, en el caso del cólera, derivaron a una terapéutica bastante amplia, que abarcaba desde inyecciones con elementos químicos hasta la ingesta de alcohol o lavados que no tenían mayores efectos en la rapidez con que actuaba el cólera.

En la primera mitad del siglo XX, los brotes de cólera fueron disminuyendo su impacto, en gran medida por la instalación de sistemas de alcantarillado, mejoras en la salubridad de las ciudades y los avances de los conocimientos para controlar infecciones. Esta tendencia duró hasta 1991-1992, cuando en Perú estalló un mortífero brote de cólera que dejó alrededor de seis mil muertes en la región. A raíz de esta situación, en la frontera norte ariqueña, y en Chile en general, se desplegaron campañas informativas, preventivas y varios dispositivos que contribuyeron a reducir los efectos de la epidemia en el país.
